# Somatic mutations in angiogenesis-related pathways and RNA polymerase II activity in sporadic brain arteriovenous malformations

**DOI:** 10.3389/fneur.2025.1660604

**Published:** 2025-09-26

**Authors:** Rebeca Pérez-Alfayate, Vanesa García-Barberán, Isabel Casado-Fariñas, Desiré Hernández-Martínez, María E. Gómez del Pulgar, Juan Pablo Castaño-Montoya, Pedro Pérez-Segura, Santiago Cabezas-Camarero

**Affiliations:** ^1^Neurosurgical Department, Hospital Universitario Clínico San Carlos, IdISCC, Madrid, Spain; ^2^Medical Oncology Lab, Hospital Universitario Clínico San Carlos, IdISCC, Madrid, Spain; ^3^Pathology Department, Hospital Universitario Clínico San Carlos, Madrid., Spain; ^4^Medical Oncology Department, Hospital Universitario Clínico San Carlos, IdISCC, Madrid, Spain

**Keywords:** brain arteriovenous malformation, angiogenesis, somatic mutation, RNA polymerase II, PI3K pathway, DNA repair, next-generation sequencing, intracranial arteriovenous malformations

## Abstract

**Background:**

Sporadic brain arteriovenous malformations (bAVMs) are rare vascular anomalies characterized by abnormal angiogenesis and direct arteriovenous shunting. While the VEGF pathway is well studied, the genetic landscape contributing to angiogenic dysregulation remains poorly defined. We aimed to characterize the mutational profile of resected bAVMs using a pan-cancer next-generation sequencing panel, with particular focus on angiogenesis-associated pathways and RNA Polymerase II activity.

**Methods:**

A descriptive analysis of clinical and molecular characteristics was conducted In formalin-fixed, paraffin-embedded tissue from the bAVM nidus. DNA was extracted and sequenced using the Oncomine Tumor Mutational Load Assay, covering 409 cancer-related genes. Variants were filtered for pathogenicity, allele frequency, and functional relevance.

**Results:**

Thirteen sporadic bAVMs were retrospectively analyzed. Twelve pathogenic variants were detected in 7/13 (54%) patients, with variant allele frequencies ranging from 3.61 to 50.61%. Most mutations clustered within angiogenesis-related pathways (PI3K/AKT/mTOR, RAS/MAPK), DNA repair mechanisms, and transcriptional regulators of RNA Polymerase II. Notably, six mutations involved genes with known functional links to RNA Pol II activity. These findings suggest a converging role for transcriptional dysregulation and vascular remodeling in bAVM pathogenesis.

**Conclusion:**

This study proposes a novel hypothesis implicating RNA Polymerase II-mediated transcription in the aberrant angiogenesis of bAVMs. While KRAS mutations were detected at low frequency and allele burden, other genetic alterations in DNA repair and transcriptional machinery may drive or sustain vascular instability. Further functional validation is warranted to clarify their pathogenic role and therapeutic potential.

## Introduction

Brain arteriovenous malformations (bAVMs) are vascular anomalies characterized by tortuous, morphologically abnormal channels that create direct connections between arteries and veins, bypassing the capillary network. This anatomical defect results in high-pressure arterial blood being shunted directly into the venous drainage system. Affecting approximately 15 per 100,000 individuals, bAVMs represent a major cause of hemorrhagic stroke, particularly in young adults (1–2).

Currently, four treatment options are available for unruptured brain arteriovenous malformations (bAVMs): microsurgical resection, radiosurgery, embolization, and conservative management. The management of unruptured bAVMs remains controversial, and treatment decisions should be guided by the patient’s clinical condition, the natural history of the disease, and the radiological characteristics of each case. Given these factors, existing treatment modalities are not sufficiently safe (1). Therefore, a deeper understanding of the pathogenesis of bAVMs, the identification of potential therapeutic targets, and the development of more personalized treatments are crucial to improve patient outcomes (2).

While the precise etiology of sporadic bAVMs remains unknown, similar vascular lesions have been observed in rare genetic syndromes (3, 4). Sporadic brain arteriovenous malformations (bAVMs) may arise from aberrant molecular signaling pathways, leading to abnormal angiogenesis. While the vascular endothelial growth factor (VEGF) pathway is the most extensively studied (5, 6), recent research suggests that high-flow bAVMs may be due to somatic mutations, affecting mainly the RAS-MAPK pathway, and especially affecting *KRAS* and *BRAF* (7–9). It has also been suggested that epigenetic changes such as methylation or hypermethylation may contribute to bAVM pathogenesis (10). On the other hand, some polymorphisms can increase the risk of bAVM rupture by elevating the expression of certain inflammatory cytokines (11).

This study aimed to characterize the mutational profile (MP) of a series of resected bAVMs to identify potentially actionable alterations.

## Materials and methods

### Patients

A retrospective series of 13 consecutively resected sporadic brain arteriovenous malformations (bAVMs) was analyzed following approval by the Institutional Review Board (IRB), in accordance with the principles outlined in the World Medical Association Declaration of Helsinki (IRB code: 23/332-E). Written informed consent was obtained from all patients prior to study participation. Clinical records were reviewed for patient demographics, presenting symptoms, and medical history, with a focus on intracranial or extracranial vascular lesions (Table 1). Imaging studies were also analyzed to define bAVM Spetzler-Martin and Lawton-Young scores. Family history was assessed for bAVMs, vascular lesions, or stroke.

**Table 1:** Clinical characteristics of patients in this study.

Age	Mean	Range	
	39.46	20–72	
Gender	Male	Female	
	53.80%	46,2%	
Hemorrhagic presentation	38.50%		5/13
Seizures	23%		3/13
Incidental	23%		3/13
Location	Frontal	61.50%	8/13
	Temporal	7.70%	1/13
	Parietal	7.70%	1/13
	Occipital	7.70%	1/13
	Cerebellum	15.40%	2/13
Spetzler Martin score	Grade I	30.80%	4/13
	Grade II	38.40%	5/13
	Grade III	30.80%	4/13
Lawton young score	3 points	7.70%	1/13
	4 points	15.40%	2/13
	5 points	46.15%	6/13
	6 points	15.40%	2/13
	7 points	7.70%	1/13
	8 points	7.70%	1/13
Prior treatment before microsurgery	Embolization	38.50%	5/13
	Radiosurgery	7.70%	1/13

### Samples and preparation

Formalin-fixed, paraffin-embedded (FFPE) tissue sections, selected by a pathologist from the bAVM nidus, were used for DNA extraction and quantification. Slides were assessed to determine tissue adequacy and viability for molecular testing. Cases were excluded if the tissue quantity was insufficient or if extensive artifact-related damage compromised sample integrity. These samples were retrospectively selected from an institutional biobank, ensuring they met quality criteria such as tissue integrity and absence of significant contamination. Prior to extraction, FFPE tissue sections were deparaffinized manually.

### DNA extraction

DNA extraction was performed using the QIAamp DNA FFPE Tissue Kit (QIAGEN, Germantown, MD, USA), specifically designed for FFPE samples where DNA may be fragmented and cross-linked due to formalin fixation. This kit employs silica-based column technology that allows selective binding of DNA to a membrane under chaotropic conditions, followed by washes to remove inhibitors such as proteins, salts, and formalin residues. The protocol involved: (1) tissue lysis with proteinase K to digest proteins and release DNA; (2) incubation at elevated temperatures (approximately 56–90 °C) to reverse formalin-induced cross-links; (3) column-based purification with specific buffers (AW1 and AW2 for washes, and AE for elution). This yields high-purity DNA suitable for downstream applications like sequencing. Multiple aliquots per sample were processed to ensure reproducibility, and over-extraction was avoided to minimize degradation.

### DNA quantification

DNA quantification was carried out using the QUBIT 3.0 fluorometer (Thermo Fisher Scientific, Waltham, MA, USA), which uses dsDNA-specific fluorescent dyes. QUBIT provides a selective and sensitive measurement (detection range of 0.2–100 ng/μL). The protocol involves mixing 1–20 μL of sample with the QUBIT dsDNA HS (high sensitivity) or BR (broad range) reagent, brief incubation, and fluorescence measurement excited at ~502 nm with emission at ~523 nm. A minimum of 20 ng of DNA per sample was required to proceed with library preparation, with adjustments to elution volume if necessary to concentrate the DNA.

### Next-Generation sequencing and mutational profiling

The mutational profile and tumor mutational burden (TMB, defined as the number of somatic mutations per megabase of coding DNA) were assessed using next-generation sequencing with the Oncomine Tumor Mutation Load Assay (Thermo Fisher Scientific, Waltham, MA, USA). This targeted panel covers 1.65 Mb of exonic and intronic regions across 409 genes frequently altered in cancer (including oncogenes such as KRAS, BRAF, PIK3CA, and tumor suppressors like TP53), optimized for detecting low-frequency somatic variants in FFPE samples with limited DNA. The assay uses AmpliSeq technology, which amplifies target regions via ultra-deep multiplex PCR, enabling uniform coverage (>95% at 500x average depth) and detection of variants with allelic frequencies (VAF) as low as 5–10%.

Library construction was automated using Chef-Ready Kits with 20 ng of input DNA, minimizing bias from manual handling. This step involved: (1) multiplex amplification of target amplicons (typically 12–24 PCR cycles to avoid artifacts); (2) partial primer digestion with FuPa reagent; (3) ligation of Ion Torrent adapters with barcodes for sample multiplexing; and (4) purification with magnetic beads (AMPure XP) to select fragments of optimal size (~200–300 bp). Libraries were loaded onto an Ion 540 chip using the Ion Chef Instrument, which performs automated emulsification and enrichment of sequencing particles (Ion Sphere Particles, ISPs) loaded with DNA. Sequencing was performed on the Ion GeneStudio S5 System (Thermo Fisher Scientific, Waltham, MA, USA), based on semiconductor sequencing technology (Ion Torrent). This method detects pH changes caused by proton release during nucleotide incorporation, eliminating the need for laser optics and enabling rapid runs (~2–4 h per chip). It was configured for single-end reads with an average length of 200 bp, achieving an average coverage depth of 500-1000x for optimal TMB sensitivity.

### Bioinformatic analysis

Raw data (BAM/FASTQ files) were analyzed using Ion Reporter version 5.12 (Thermo Fisher Scientific, Waltham, MA, USA), a cloud-based platform for automated processing of Ion Torrent data. The Coverage Analysis plugin was used to assess coverage uniformity, read quality (Phred score >20), and metrics such as the percentage of on-target bases (>90% expected). The specific workflow “Oncomine Tumor Mutation Load-w3.4-LOD0.1” was applied for variant calling, incorporating alignment to the hg19/GRCh37 reference genome, filtering of artifacts (e.g., homopolymers common in Ion Torrent), and TMB calculation. The limit of detection (LOD) of 0.1 indicates sensitivity for variants with allelic frequency ≥10%, adjusted for background noise in FFPE samples.

Variant allele frequency (VAF) was calculated as the proportion of reads supporting the variant allele divided by the total reads covering that genomic position, expressed as a percentage. A reporting threshold of ≥5% VAF was applied in line with the validated sensitivity limits of the Oncomine assay.

Variants were annotated using “Oncomine Tumor Mutation Load Assay Annotations v1.5,” which integrates databases like COSMIC, dbSNP, and 1,000 Genomes for functional context (e.g., synonymous, nonsynonymous, frameshifts). The “Oncomine Variants (5.20)” filter was applied to prioritize cancer-relevant variants, excluding common polymorphisms (MAF > 1%) and technical artifacts. Each gene variant was classified manually or semi-automatically using the ClinVar database,^1^ a NIH-curated repository providing evidence-based clinical interpretations. Variants were categorized as pathogenic if classified as “pathogenic” or “likely pathogenic.” Non-pathogenic variants included: (1) “likely benign” or “benign,” based on lack of functional impact; (2) variants of uncertain significance (VUS), where evidence is insufficient; and (3) those not documented in ClinVar, considered benign by default unless additional functional analyses (e.g., in silico with SIFT/PolyPhen) suggested otherwise. Cross-validation with tools like Variant Effect Predictor (VEP) was performed if needed to resolve ambiguities.

## Results

### Patients

Among the 13 patients included in the study, six were female and seven were male. The mean age was 39.5 years (range: 20–72 years). The most common clinical presentation was intracranial hemorrhage (5/13, 38.5%), followed by seizures (3/13, 23.1%) and incidental findings (3/13, 23.1%); less frequent presentations included headache and cerebellar ataxia. No patient had relevant comorbidities. Data related to the angioarchitectonic characteristics of the bAVMs are summarized in Table 1. All patients had a surgical indication for bAVM. Preoperative embolization was required in five cases due to the presence of flow-related aneurysms or acute bleeding. One patient had previously undergone stereotactic radiosurgery (SRS), which failed to achieve complete bAVM closure. The lowest TMB was observed in those cases that had undergone prior embolization (Table 2).

**Table 2:** Summary of the cases and the pathogenic mutations found in the study.

Case	Gender	Age	Spetzler-Martin score	Lawton and young score	Hemorrhagic presentation	Clinical presentation	Location	Laterality	Previous treatment of the AVM	TMB (mutations/Mb)	Pathogenic Genes found	Type	Variant Effect	Allele frequency % (VAF)	Mutation	Amino acid change
1	Male	37	III	5	Yes	ICH	Cerebellum	Right	No	3.38	ERCC2	INDEL	Frameshift Insertion	40.52	c.1793_1796dup	p.Ala600SerfsTer50
											SOX11	SV	Missense	3.95	c.151C > T	p.Arg51Trp
											MTRR	INDEL	Nonsense	3.61	c.340C > T	p.Arg114Ter
2	Female	35	I	4	Yes	ICH	Temporal	Right	No	1.7	ND	ND	ND	ND	ND	ND
3	Female	20	I	3	No	Seizures	Frontal	Left	No	20.08	KRAS	SNV	Missense	9.41	c.35G > A	p Gly12Asp
											MUTYH	SNV	Missense	3.80	c.722G > A	p Arg241Gln
											ATM	SNV	Missense	4.41	c.9023G > A	p.Arg3008His
											G6PD	SNV	Missense	48.33	c.466A > G	p Asn156Asp
4	Male	56	II	5	No	Incidental	Frontal	Right	No	9.24	FH	SNV	Missense	3.66	c.1202G > A	p.Gly401Glu
5	Male	66	II	5	No	Headache	Frontal	Left	No	2.53	ND	ND	ND	ND	ND	ND
6	Male	38	II	5	Yes	ICH	Frontal	Right	Embolization	1.69	ND	ND	ND	ND	ND	ND
7	Female	30	III	6	No	Seizures	Parietal	right	Embolization	0.85	ND	ND	ND	ND	ND	ND
8	Male	41	II	5	Yes	ICH	Occipital	Right	No	1,7	ND	ND	ND	ND	ND	ND
9	Female	47	III	7	No	Incidental	Frontal	Left	Embolization	0.85	TAF1	SNV	Missense	5.41	c.4270C > T	p.Arg1424Trp
10	Male	41	III	8	No	Seizure	Frontal	left	SRS Embolization	1.69	ND	ND	ND	ND	ND	ND
11	Female	52	I	4	No	Cerebellar ataxia	Cerebellum	Right	No	1.7	PIK3R2	SNV	Missense	3.83	c.1117G > A	p.Gly373Arg
12	Male	72	II	6	Yes	ICH	Frontal	left	Embolization	0.85	KMT2D	SNV	Nonsense	3.90	c.14878C > T	p.Arg4960Ter;
13	Female	66	I	5	No	Incidental	Frontal	left	No	1.7	ERCC1	SNV	Missense	50.66	c.693C > G	p.Phe231Leu

ICH, intracerebral hemorrhage; INDEL, insertion–deletion; ND, not determined; SNV, Single nucleotide variant.

### Mutational analysis

**Figure 1:**
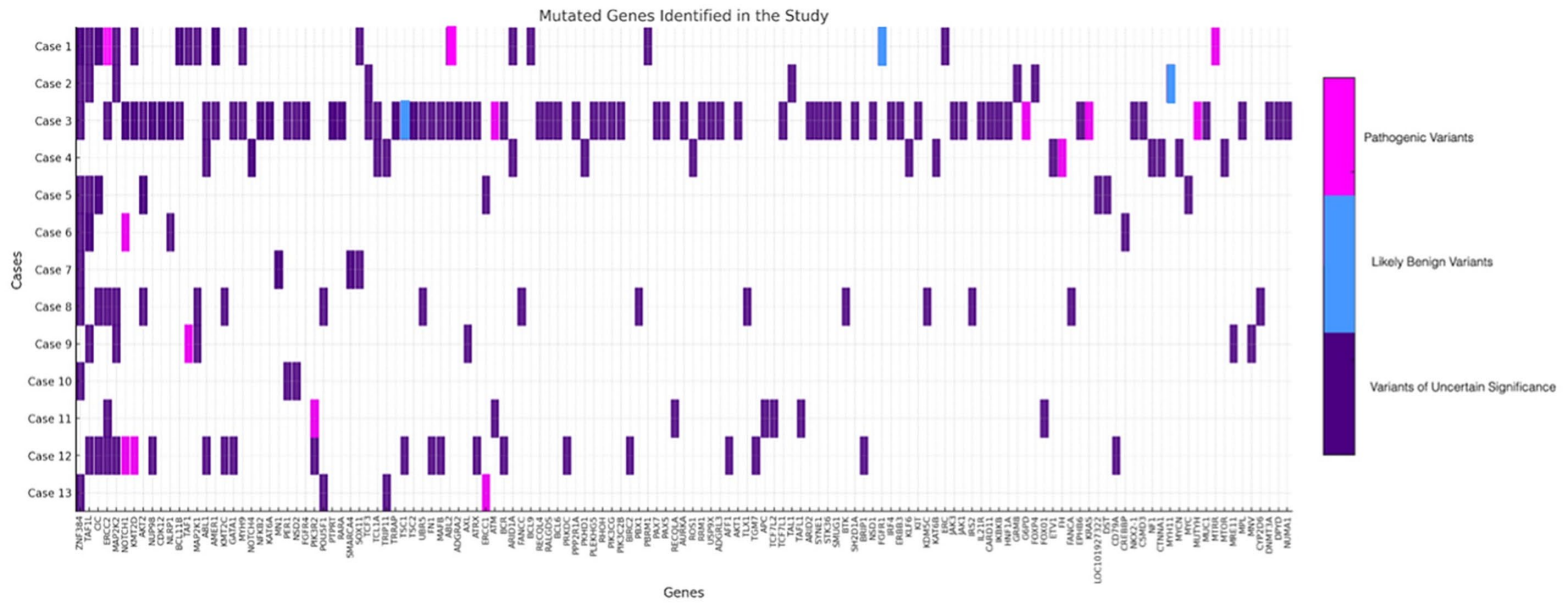
Schematic representation of all variants with an allele frequency of approximately 5% or higher. Variants of uncertain significance are depicted in purple, likely benign variants in blue, and pathogenic variants in pink.

Following next-generation sequencing analysis. Within the panel of 409 analyzed genes, 224 mutations were identified. Among these, 12 genes harbored pathogenic variants (Figure 1).

The TMB ranged from 0.85 to 20.08 mutations per megabase. The analysis of mutation frequency within the sample revealed a heterogeneous distribution of genetic alterations across multiple genes.

The 12 pathogenic variants were identified in seven out of the 13 patients. Allele frequencies (VAF) ranged from 3.61 to 50.61%, suggesting a somatic origin. In case 1, pathogenic variants were detected in ERCC2 (c.1793_1796dup; VAF: 40.52%), SOX11 (c.151C > T; VAF: 3.95%), and MTRR (c.340C > T; VAF: 3.61%). Case 3 exhibited mutations in KRAS (c.35G > A; VAF: 9.41%), MUTYH (c.722G > A; VAF: 3.80%), ATM (c.9023G > A; VAF: 4.41%), and G6PD (c.466A > G; VAF: 48.33%). In case 4, a pathogenic variant was identified in FH (c.1202G > A; VAF: 3.66%). Case 9 presented a mutation in TAF1 (c.4270C > T; VAF: 5.4%), while case 11 exhibited a pathogenic variant in PIK3R2 (c.1117G > A; VAF: 3.83%). Additionally, case 12 carried a mutation in KMT2D (c.14878C > T; VAF: 3.90%), and case 13 harboured a pathogenic variant in ERCC1 (c.693C > G; VAF: 50.66%; Table 2).

**Figure 2:**
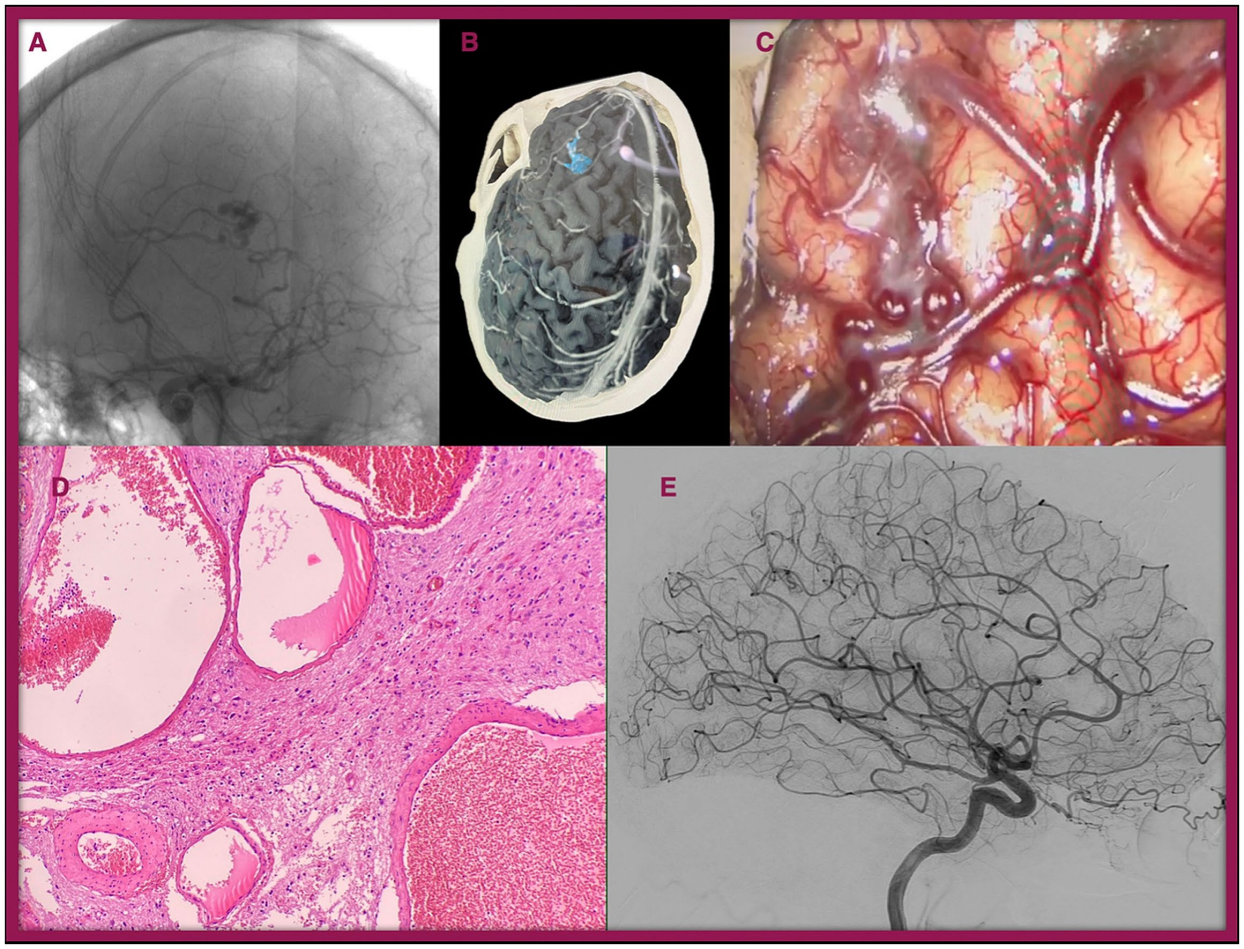
Illustration of Case 3. A 20-year-old female presented with seizures. During the workup, a bAVM (Spetzler-Martin grade I, Lawton-Young 3) was identified in the left frontal lobe. **(A)** Preoperative conventional angiogram. **(B)** 3D reconstruction of the lesion. **(C)** Intraoperative image of the lesion. **(D)** Histopathological view with Hematoxylin–Eosin staining (×40): Cluster of arterial and venous vessels with dilated lumens lined by mature endothelium, lacking an intervening capillary bed, and associated with brain parenchyma showing reactive gliosis. **(E)** Postoperative angiogram showing complete resection of the bAVM.

It is worth mentioning Case 3 which involved a 20-year-old woman with no relevant personal or family medical history, diagnosed with a Spetzler-Martin I, Lawton-Young 3 bAVM. The patient initially presented with a seizure, prompting further investigation. The case is illustrated in Figure 2.

## Discussion

This study reveals a potentially novel convergence of pathogenic mutations affecting angiogenesis, DNA repair, and transcriptional regulation via RNA Polymerase II (Pol II) in sporadic bAVMs. While previous research has identified somatic mutations in *KRAS* and *BRAF* as potential drivers of vascular malformations (7–9). Our results suggest that the mutational landscape of bAVMs is broader and functionally interconnected. The detection of mutations in genes related to transcriptional machinery and genome integrity introduces a more complex model of disease pathogenesis that extends beyond canonical angiogenic pathways.

### Molecular heterogeneity and the role of KRAS in bAVM pathogenesis

Our cohort included mostly low-grade (Spetzler-Martin I–II) bAVMs, which reflects the surgical selection bias common in most tissue-based studies (9). Haemorrhagic presentation was present in 38.5% of cases, consistent with natural history data (12). The lack of high-grade lesions limits the generalizability of our findings, as these bAVMs may exhibit a different molecular signature. This limitation is shared by previous studies, such as that by Tao-Hong et al. (9) which included only one Spetzler-Martin IV case. Alternative tissue-sampling techniques, such as liquid biopsy, have been proposed but remain limited in sensitivity. Nikolaev et al. (7) for instance, failed to detect *KRAS* mutations in paired plasma samples from patients with *KRAS*-positive nidus tissue. Endoluminal biopsy, recently demonstrated by Winkler et al. (13) in four bAVM cases, may offer a minimally invasive way to sample tissue from high-grade or unresectable lesions *in vivo*.

We detected a *KRAS* mutation in only 1 of 13 patients (7.7%), a much lower rate than previously reported by Nikolaev et al. (7) (62.5%) and Tao-Hong et al. (9) (up to 87.1% including *BRAF*). These discrepancies likely reflect differences in sequencing technology and sensitivity. Our study used a pan-cancer amplicon-based panel optimized for tumor mutational burden (TMB), with a ~ 5% variant allele frequency (VAF) detection limit. In contrast, Nikolaev et al. used whole-exome sequencing with ~100 × −200 × coverage (7), while Tao-Hong et al. combined panel Next Generation Sequencing with ddPCR validation and ultra-deep sequencing (>1,000×), enabling detection of subclonal mutations with lower VAF (9).

This raises the question of whether low-VAF *KRAS* mutations are merely passenger mutations or true drivers of vascular dysregulation. Although our results support a broader mutational landscape, the biological relevance of *KRAS* cannot be discounted. As a dominant oncogene, even subclonal *KRAS* mutations may exert strong downstream effects on MAPK signaling and angiogenesis. In cancer and other vascular malformations, low-frequency oncogenic mutations have been shown to act as early drivers that expand under selective conditions (14). Tao-Hong et al. (9) found an inverse correlation between VAF and nidus size, further suggesting a possible growth-promoting role for early *KRAS*/*BRAF* events. Conversely, Al-Olabi et al. (15) demonstrated in a zebrafish model that expression of *BRAFV600E* alone caused vascular dysplasia in only 10–20% of cases, supporting a two-hit model in which an initial mutation sets the stage for further disruption. Our identification of multiple co-occurring mutations in angiogenic, DNA repair, and metabolic genes—particularly in Case 3—suggests that *KRAS* may act in concert with other lesions to promote lesion development and progression. In addition, the overall mutational profile in our cohort was highly heterogeneous, with most variants occurring in single cases. The fact that only one patient harbored a KRAS mutation, in contrast to prior reports of recurrent KRAS alterations, underscores the exploratory nature of our findings and highlights the need for cautious interpretation.

Importantly, this interpretation is reinforced by recent endothelial models demonstrating that somatic activation of KRAS or BRAF in vascular endothelium is sufficient to induce AVM formation, with MEK/ERK identified as the critical downstream effector pathway (16). These preclinical findings strengthen the biological plausibility of our observations and highlight the translational potential of pathway-targeted therapies.

### Beyond angiogenesis: DNA repair, transcriptional dysregulation, and pol II pathways

In addition to *KRAS*, we identified 12 pathogenic variants across genes involved in angiogenesis (e.g., *PIK3R2*, *SOX11*, *KRAS*) (17–19). DNA repair (*ERCC2*, *ERCC1*, *ATM*, *MUTYH*, *G6PD*, *FH*) (20–25). DNA transcription (*TAF1*) (26, 27). and epigenetic modulation (*KMT2D*, *MTRR*) (28–30). Notably, several of these genes intersect with RNA Polymerase II (Pol II) function (*ERCC2*, *ATM*, *KRAS*, *G6PD*, *TAF1*, *KMT2D*), a transcriptional hub that mediates angiogenic signaling downstream of VEGF, KRAS-MAPK, and HIF-1α (26, 27). While Pol II is not typically viewed as an angiogenic regulator per se, its disruption could impair endothelial gene expression programs and promote abnormal vessel formation. To our knowledge, this connection between Pol II dysfunction and bAVMs has not been previously described. However, this proposed link remains hypothetical, as our study did not include functional assays to confirm pathway activation. Therefore, the role of Pol II dysfunction in AVM pathogenesis should be interpreted as exploratory and will require validation in future cellular and animal models.

Further supporting a developmental transcriptional dysregulation model, recent single-cell RNA-sequencing of human brain vasculature demonstrated reactivation of embryonic gene programs in bAVM endothelial cells (31). Our findings align with this notion, suggesting that genetic lesions affecting chromatin remodelers (*KMT2D*), DNA repair factors (*ATM*, *MUTYH*), and Pol II regulators (*TAF1*) may collectively produce a vascular phenotype that retains fetal-like characteristics and abnormal angiogenic responsiveness.

Taken together, these observations raise the hypothesis that alterations in DNA repair, transcriptional regulation, and angiogenic pathways could converge to create a permissive environment for AVM development. Defective DNA repair may facilitate genomic instability, while dysregulated transcriptional programs could amplify abnormal endothelial responses to angiogenic cues. These combined alterations may not act in isolation, but rather interact to promote aberrant vascular remodeling. Such a model suggests that bAVMs may arise from the interplay of multiple disrupted pathways, extending beyond canonical angiogenesis alone.

### Toward a network model of vascular instability

The interplay of DNA repair, oxidative stress, and angiogenesis becomes especially evident in Case 3, which carried mutations in *KRAS*, *ATM*, *MUTYH*, and *G6PD*. These genes converge functionally on the cellular response to oxidative stress and genomic instability (17, 18, 20–24, 32). *MUTYH* is critical in base-excision repair of oxidative lesions (33), *ATM* regulates DNA damage checkpoints (21), and *G6PD* controls the redox balance through NADPH generation (23). Disruption in these pathways may promote secondary oncogenic events, such as *KRAS* activation, and create a permissive environment for clonal expansion. Such cases support a network model of pathogenesis, in which no single mutation is sufficient, but together they impair vascular stability and remodelling.

Notably, Case 3 was also the youngest patient in our series (20 years old), raising the hypothesis that higher mutational burden could be linked to earlier clinical onset. This is consistent with prior observations that pediatric and young-adult AVMs often exhibit distinct clinical behavior, including higher recurrence rates after treatment. Hak et al. (34) conducted a meta-analysis showing an overall recurrence rate of 10.9% in pediatric patients, with recurrence risk decreasing significantly with each additional year of age at diagnosis (RR 0.97, 95% CI 0.93–0.99; *p* = 0.046).

This concept has therapeutic implications. Bevacizumab, an anti-VEGF agent, showed modest clinical effects in a small pilot study of two bAVM patients conducted by Muster et al. (35). Our findings suggest that targeting VEGF alone may not be sufficient, as the dysregulation extends beyond classic angiogenic signaling. Intervening in transcriptional regulation, DNA repair, or redox homeostasis may be needed to fully correct the molecular imbalance. As summarized in Table 3, several of the pathogenic variants identified in our cohort affect genes that are already known targets—or are mechanistically linked to targets, of approved or investigational drugs, including inhibitors of KRAS [e.g., adagrasib (36), sotorasib (36, 37)], PI3K [e.g., alpelisib (29), duvelisib (28)], ATM (e.g., imatinib), and epigenetic modulators (e.g., entacapone). This highlights the translational relevance of our mutational profiling and warrants further validation in preclinical models and single-cell profiling studies.

**Table 3:** Altered pathways and potential targeted therapies for each of the pathogenic gene variants detected.

Pathogenic Gene	Function	Pathways	Drugs that could potentially target the gene or the pathway	Mechanism of action of the drug
ERCC2	DNA repair	DNA repair mechanism. Nucleotide scission repair (41). RNA Polymerase II transcription initiation and promoter clearance (42) Transcription-Coupled nucleotide excision repair (TC-NER) pathway (43).	Cisplatin (43)	Inhibits DNA synthesis
			Paclitaxel (44)	Promotes assembly and inhibits disassembly of microtubules. Microtubulin disassembly inhibitor,
SOX11	Transcription factor. Transcriptional activator	ERK signaling, SOX11/FAK/ PIK3 axis (45)	FAK- and CXCR4-specific inhibitors (45)	Block SOX11 activation (45)
MTRR	DNA methylation	Cobalamin metabolism (46) Apoptosis and autophagy pathways (47)	-	-
MUTYH	DNA repair	Base excision repair (48). Packing of telomere ends (49).	-	-
ATM	DNA damage sensor	Signal transduction for the DNA damage response, apoptosis, senescence and DNA pathways (50) RNA Polymerase II transcription (51)	Imatinib (52)	Inactivation of ATM/ATR signaling
KRAS	Regulation of cell proliferation. Induce transcriptional silencing of tumor suppressor genes. Angiogenesis	MAPK/ERK pathway (53) RNA Polymerase I and II transcription pathway (33, 36, 54)	Adagrasib (36)	KRAS inhibitor
			Sotorasib (36, 37)	KRAS inhibitor
G6PD	Metabolic function	Reduction of NADPH leading to an antioxidant or a pro-oxidant environment which can enhance DNA oxidative damage (32). Involved in MTOR signaling (17). ATM signaling pathway (18) RNA Polymerase II transcription (19)	Chloroquine (20)	-
FH	DNA repair Metabolic function	DNA repair (21) TCA cycle (22)	Bevacizumab + erlotinib (22)	Anti-VEGF + epidermal growth factor receptor (EGFR) tyrosine kinase inhibitor (TKI) class
TAF1	DNA transcription	RNA Polymerase II transcription (23) MAP kinase signal transduction pathway (24, 25)	Doxorubicin (26)	Inhibits DNA topoisomerase II
PIK3R2	Activates signaling cascades involved in cell growth, survival, proliferation, motility and morphology Angiogenesis	AMPK signaling and PI3K-AKT pathway (27)	Duvelisib (28)	PI3K-δ/PI3K-γ inhibitor
			Alpelisib (29)	Selective PI3Kα inhibitor, Kinase Inhibitors, PI3K/MTOR Dual Inhibitor.
KMT2D	Histone methyltransferase	Gene expression (transcription) RNA polymerase II transcription (30, 55)	Entacapone (56)	COMT inhibitor
ERCC1	DNA repair	Transcription-Coupled nucleotide excision repair (TC-NER) pathway (57).	Carboplatin (58)	Antitumor agent that forms platinum-DNA adducts

### Limitations

This study has several limitations. First, the small cohort and the fact that all cases were Spetzler–Martin grade I–III surgically resected bAVMs limit the generalizability of our findings to higher-grade lesions (38–40). In addition, no pediatric patients were included in this series, which may limit extrapolation of our findings to younger populations, as pediatric AVMs have been associated with distinct clinical behavior and higher recurrence rates after treatment.

Second, although the use of a pan-cancer sequencing panel could be perceived as a limitation due to its design focus on oncogenic mutations, this approach is, in fact, strategically justified in the context of bAVMs. Currently, there are no Next generation sequencing panels specifically optimized for the genetic study of sporadic brain arteriovenous malformations. Therefore, using a broad, oncology-based panel offers the advantage of covering many of the genes already implicated in bAVM pathogenesis. Notably, somatic mutations in *KRAS*, *BRAF*, and *PIK3R2*, all well-established oncogenes, have been repeatedly reported in sporadic bAVMs (7, 9, 36). These genes play central roles in angiogenesis-related signaling pathways, including RAS-MAPK and PI3K-AKT, which are essential to both tumor biology and vascular development. In this sense, the pan-cancer panel serves not only as a pragmatic solution in the absence of a bAVM-specific tool, but also as a biologically relevant platform to explore the somatic landscape of these lesions. Nevertheless, we acknowledge that the pathogenic relevance of the detected variants remains uncertain, and our results should be interpreted as exploratory and hypothesis generating rather than definitive.

Third, the panel’s 5% VAF threshold likely missed subclonal variants detectable only through ultra-deep or ddPCR-based approaches (8). Fourth, lack of functional validation (e.g., protein expression, pathway activation) precludes mechanistic conclusions.

Finally, although functional validation (e.g., protein expression, pathway activation) was not performed in this study, we view this not solely as a limitation but as a critical avenue for future research. Functional studies in cellular and animal models will be essential to confirm the mechanistic contribution of these mutations and to assess their potential as therapeutic targets. In addition, the restricted gene coverage of the panel and the absence of recurrently mutated genes across patients further limit the strength of our conclusions, underscoring that these findings should be considered exploratory and hypothesis-generating. Moreover, patient heterogeneity in treatment history (embolization, radiosurgery) could introduce confounding.

## Conclusion

Our findings support the presence of a complex mutational profile in sporadic brain AVMs, with convergence on angiogenesis, DNA repair, and RNA Polymerase II-mediated transcription pathways. The identification of multiple mutations associated with Pol II function suggests a novel mechanism of vascular dysregulation, potentially linking genetic and epigenetic signals to aberrant vessel formation.

Although KRAS mutations were infrequent and low in allele frequency, other functionally relevant alterations may contribute to a broader molecular network underlying bAVM pathogenesis. These insights provide a framework for future studies exploring transcriptional regulation in AVMs and open the door for potential therapeutic interventions targeting these pathways.

## Data Availability

The datasets generated and analyzed for this study are contained within the article. Additional anonymized data underlying the findings of this study are available from the corresponding author upon reasonable request, in accordance with institutional and ethical guidelines.

## Footnotes

^1^ https://www.ncbi.nlm.nih.gov/clinvar/
